# Visual detection of cyanide using ninhydrin coated paper

**DOI:** 10.1016/j.heliyon.2025.e42283

**Published:** 2025-01-24

**Authors:** Syafril Hidayat, Rachadaporn Benchawattananon, Lapatrada Taemaitree

**Affiliations:** Department of Integrated Science, Forensic Science Program, Faculty of Science, Khon Kaen University, Khon Kaen, 40002, Thailand

## Abstract

Humans are often exposed to cyanide through drinking water or by eating plants that contain cyanogenic glucosides (e.g. cassava – a staple source of carbohydrates). Many methods exist to detect cyanide, but few are safe, cheap and easy to perform for an untrained user. In this work, we demonstrate that Whatman paper can be coated with ninhydrin and that the addition of basic solutions of cyanide gives an immediate pale yellow to red colour change. The ninhydrin paper can be used to visually, semi-quantitatively detect cyanide at concentrations up to ∼5 μg/mL (in 30 μL; 0.15 μg), and can be stored for several months. Most importantly, we show the paper can be used to monitor the release of cyanide from plants such as cassava leaves as they are processed in cooking suggesting the paper could be used in-the-field.

## 1 Introduction

Cyanide is an extremely toxic anion. It has a high affinity for metal ions and binds tightly to iron in cytochrome oxidase enzymes. This disrupts the body's electron transport chain preventing aerobic generation of energy (ATP) [1]. In nature, the cyanide ion or its protonated form can be found in the air, water and food. For example, industrial processes such as gold mining or plastic production release cyanide in large quantities [2], while numerous plants contain cyanogenic glucosides that release cyanide upon consumption [3]. Exposure to low levels of cyanide can cause dizziness, nausea or vomiting, while high levels can cause death. As of 2022, the World Health Organisation's (WHO) reported a highly conservative health-based exposure limit of 0.5 μg/mL over 5 days (2 L per day; i.e. 1 mg per day for 60 kg body weight) [4], while the US Environmental Protection Agency (US EPA) limit from 1992 is 0.2 μg/mL (which is “the level at which no known or anticipated adverse effects on the health of persons occur and which allows an adequate margin of safety”) [5].

Numerous small molecules and analytical techniques exist to determine cyanide concentrations [[6], [7], [8], [9]], but few are capable of doing this in purely aqueous environments [10,11], and fewer still are simple to perform [12,13]. In order to detect cyanide, skilled operators of complex machinery are often required. There are a handful of validated laboratory-based methods that are routinely used to determine cyanide concentrations (e.g. chromatography, voltammetry and titrations) [14]. However, not all methods need to be quantitative. Given that people can be frequently exposed to cyanide, there is still the need for cheap, simple and sensitive methods to detect cyanide. For example, cassava leaves and roots are eaten by over 800 million people and it is the third largest source of calories in the world: one person can consume as much as 800 g of cassava per day [15]. Yet without adequate processing [16], cyanide in cassava leaves can be as high as 1300 μg/g. Similarly, apricot kernels and lima beans can contain up to 5000 μg/g of cyanide [17]. To this end, visual colourimetric assays are critical.

Paper strip tests have been used extensively from the simple pH paper to more complex lateral flow tests that are used in pregnancy and COVID-19 tests. For cyanide, several paper-based tests exist [[18], [19], [20]]. They can detect cyanide at or below ∼1 μg/mL (typically ∼5 mL), but they have a range of limitations which means no one method is perfect. For example, Cyantesmo requires the use of concentrated sulphuric acid that evolves gaseous inhalable hydrogen cyanide; Quantofix requires toxic reagents; Visocolor Eco is more susceptible to interferents (e.g. bromide or iodide); acidification of picrate paper can generate picrate acid that is explosive when dry; and a newer origami paper method uses non-commercial receptors to detect cyanide. For a full list of sensitivity and limitations see Supplementary Information Table 1.

Herein, we report the use of paper impregnated with non-toxic, commercially available ninhydrin to detect cyanide. The preparation of the paper is trivial and gives a pale yellow to red colour change upon the addition of as little as ∼5 μg/mL of cyanide. While the colour does fade over time, it turns purple after a couple of hours. Moreover, we demonstrate that the paper can be used to monitor the levels of cyanide in cassava leaves as they are processed to remove cyanide.

## 2 Results and discussion

**Fig. 1:**
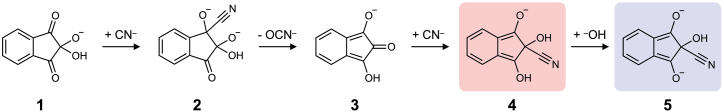
The proposed reaction of cyanide with ninhydrin (1) that enables the formation of hydrindantin (3) and a *2H* indene complex. Single (4) or double (5) deprotonation have been proposed to cause the red and blue colours observed in the reaction.

Ninhydrin is a non-toxic chemical that is widely used for chemical analysis. It has previously been used to quantify cyanide in aqueous solutions [[21], [22], [23], [24]]. Mechanistic studies suggested that cyanide reacts with ninhydrin (1) to form hydrindantin (3), which in turn reacts with another molecule of cyanide to form a stabilised *2H* indene complex that is red if singly deprotonated (4) or blue if doubly deprotonated (5) (Fig. 1) [24]. When this reaction occurs in solution, exposure of the complex to oxygen results in rapid oxidation and colour loss. Interestingly, Drochioiu et al. suggested 4 is more stable in the solid state but no further studies have been reported. As a consequence, we hypothesised that performing the ninhydrin–cyanide reaction on a solid surface may give a complex that is more stable than in solution and overcome the oxygen sensitivity of the reaction. Moreover, given that ninhydrin is used extensively in analytical chemistry (e.g. thin layer plate visualisation), biochemistry (e.g. amino acid quantification) [25] and forensic science (e.g. fingerprint detection) [26], we felt a paper-based ninhydrin assay would provide a cheap way of detecting cyanide in solution.

**Fig. 2:**
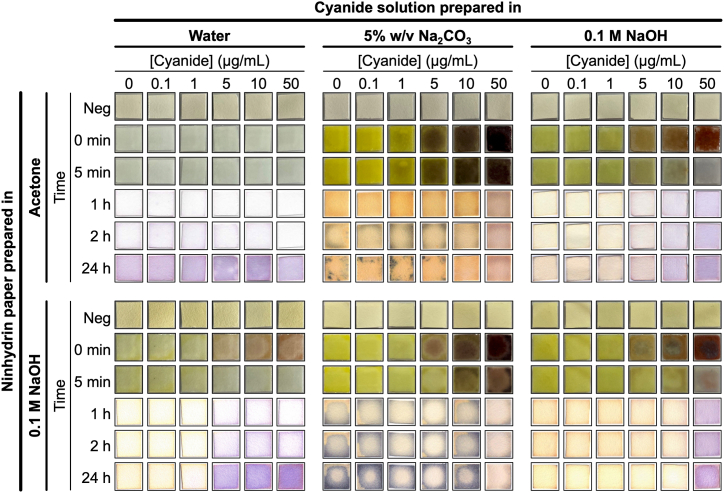
Optimisation of cyanide detection on ninhydrin paper. The Whatman paper was prepared by submerging the paper in either an acetone or a 0.1 M NaOH solution of ninhydrin and allowed to dry. Potassium cyanide (0, 0.1, 1, 5, 10 and 50 μg/mL) was prepared in either unbuffered water, 5% w/v Na_2_CO_3_ or 0.1 M NaOH, and 30 μL was added to the paper. Colour changes were recorded before (negative control) and after addition of the cyanide solution to the paper (immediately [0 min], 5 min, 1 h, 2 h and 24 h). For repeats, see Supplementary Information Fig. 1.

When designing the assay, we focused on 1) the preparation of the ninhydrin impregnated Whatman paper, 2) the pH of the cyanide solution, and 3) the effect of time on any colour changes. Ninhydrin is slightly soluble in water and more soluble in organic solvents such as acetone. For eventual use in-the-field, we recognised that a high pH is necessary for the ninhydrin–cyanide reaction to occur. As a result, we evaluated the effect of neutral or basic ninhydrin paper (prepared by dissolving ninhydrin in acetone or 0.1 M NaOH respectively). Similarly, we treated these papers with various concentrations of cyanide (0, 0.1, 1, 5, 10 and 50 μg/mL) in either unbuffered, mildly basic (5% w/v Na_2_CO_3_) or more strongly basic water (0.1 M NaOH). Finally, acknowledging that any colour changes may fade over time due to oxidation of the *2H* indene complex, we also captured images of the paper over time (before, immediately [0 min], 5 min, 1 h, 2 h and 24 h after cyanide addition). From this matrix of conditions (Fig. 2), several conclusions were reached.

Firstly, up to 5 μg/mL (in 30 μL; 0.15 μg) of cyanide can be detected by ninhydrin impregnated paper. After immediate addition of cyanide, a red colour was observed that over time becomes purple. This colour change was more intense with higher cyanide concentrations. Interestingly, while solution-based assays are pH dependent, these colour changes are time dependent: the red colour disappears after several minutes and is replaced by a purple colour after a couple of hours that is stable even 24 h later.

Secondly, high pH is still important. The most consistent and clearest colour differences were observed when either cyanide was dissolved in more strongly basic 0.1 M NaOH or the paper was prepared in a 0.1 M NaOH solution. Acetone paper was far simpler to prepare; the paper was simply submerged in a solution of acetone and ninhydrin and allowed to evaporate over ∼1 h. On the other hand, 0.1 M NaOH paper took overnight to dry and gave uneven coverage as observed by the darker staining of the edges of the paper. As a consequence, we suggest that cyanide containing samples should be pre-diluted in NaOH and added to acetone paper. This is also more practical as solutions containing cyanide are unlikely to have a consistent pH.

Thirdly, NaOH gave clearer colour changes than Na_2_CO_3_ at longer time points. For NaOH solutions, a clear pale yellow to purple colour change is observed that is dependent on cyanide concentration. On the other hand, for Na_2_CO_3_ solutions there is no clear cyanide dependent colour change with the paper remaining black/orange. Finally, we note that the concentration of ninhydrin in acetone was also evaluated (Supplementary Information Fig. 2) and that higher concentrations gave clearer red/purple colour changes as expected.

In summary, the best condition for the assay was the use of an acetone solution of ninhydrin to prepare the paper and the addition of cyanide in a 0.1 M NaOH solution. A clear pale yellow to red colour change was observed that became purple over serval hours and remained this colour even 24 h later. We also confirmed that the paper can be stored for several months in a dark bottle with minimal differences in colour change compared to a freshly prepared batch of ninhydrin paper in acetone (Supplementary Information Fig. 3). These conditions are used in the remainder of this work.

**Fig. 3:**
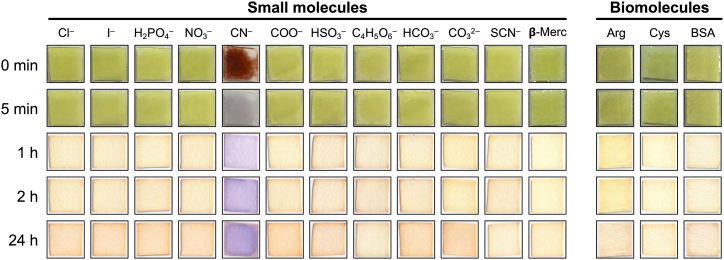
Ninhydrin paper is specific for cyanide over other molecules such as thiols (**β**-Merc = **β**-Mercaptoethanol), amino acids (arginine and cysteine) and proteins (BSA = bovine serum albumin). Concentration = 50 μg/mL. Volume added = 30 μL. Colour changes were recorded immediately (0 min), 5 min, 1 h, 2 h and 24 h after addition of each solution onto the paper. Note all molecules are dissolved in 0.1 M NaOH.

Having established the optimal conditions, we focused on the specificity of the assay. Previous reports indicate the ninhydrin–cyanide reaction is selective for cyanide in solutions [22], and we confirmed this is also true for the reaction on paper. Of the various molecules tested (30 μL of 50 μg/mL of Cl^−^, I^−^, H_2_PO_4_^−^, NO_3_^−^, COO^−^, HSO_3_^−^, C_4_H_5_O_6_^−^, HCO_3_^−^, CO_3_^2−^, SCN^−^ and **β**-Mercaptoethanol) including structurally similar thiocyanate (SCN^−^), only cyanide gave a characteristic pale yellow to red/purple colour change (Fig. 3). Furthermore, other biomolecules that may be present in real world samples such as amino acids (e.g. arginine or cysteine) or proteins (e.g. bovine serum albumin [BSA]) failed to generate a positive colour change. For sulfur containing molecules that are more likely to react with ninhydrin (HSO_3_^−^, SCN^−^, **β**-Mercaptoethanol, cysteine and BSA), we confirmed that even higher concentrations did not elicit a positive reaction (10-fold higher; Supplementary Information Fig. 4). These results were encouraging because real world samples are likely to contain lower concentrations of interferents than the concentrations we have tested.

Cassava roots and leaves are widely consumed and must be processed to reduce cyanide levels [16,27]. Cassava leaves contain more cyanide than roots and are also naturally more nutritious. A common method to process cassava leaves is to crush them thereby releasing the glucosidase linamarase to break down the cyanogenic glucoside linamarin to glucose and hydrogen cyanide. Once crushed, the leaves are either boiled to enhance the breakdown and release of cyanide, or left to stand in water to facilitate the enzymatic reaction. The supernatant is then removed and the process is repeated until cyanide levels are low enough [28].

**Fig. 4:**
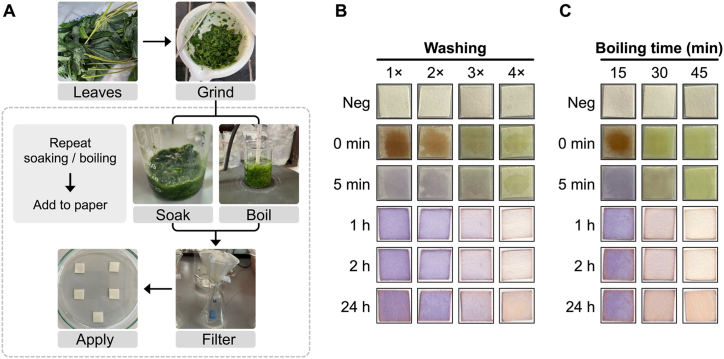
Detection of cyanide in cassava leaves using ninhydrin paper. (A) shows a schematic of the process. Cassava leaves were crushed and either washed (soaked) or boiled in water. The supernatant after every 15 min of each process was then diluted 10-fold in a 0.1 M NaOH solution and added to ninhydrin paper. The process was repeated until the amount of cyanide is negligible. (B) shows colour changes after washing once (1×), twice (2×), three times (3×) and four times (4×). (C) illustrates colour changes after boiling for 15 min, 30 min and 45 min. Colour changes were recorded before (negative control) as well as immediately (0 min), 5 min, 1 h, 2 h and 24 h after addition of the cyanide solution to the paper. As expected, more washes and longer boiling with water resulted in lower cyanide concentrations. For repeats, see Supplementary Information Fig. 7. Cassava leaves were obtained from Khon Kaen, Thailand.

To evaluate, the ability of the ninhydrin paper to detect cyanide in real samples, we used these cassava pre-processing methods and applied the supernatant to the ninhydrin paper to monitor the process. The supernatant was diluted 10-fold in 0.1 M NaOH before application to the paper. Pleasingly, the initial supernatant contained a high level of cyanide and was easily visualised by our ninhydrin paper method (Fig. 4). The supernatant from subsequent steps showed lower amounts of cyanide until the third boiling step or the fourth washing step, which showed no cyanide. These results are consistent with what has been reported previously for cassava processing [28].

We confirmed our findings by first spiking the supernatant with exogenous cyanide. This demonstrated the expected colour changes are observed in a complex media when cyanide is present (Supplementary Information Fig. 5). Secondly, we quantified the amount of cyanide in the cassava leaf extracts using the solution phase ninhydrin method (Supplementary Information Fig. 6). This confirmed that the paper colour changes correspond roughly to those observed using cyanide only solutions (∼10 μg/mL, 30 μL; Fig. 1). Finally, we performed a completely independent analysis on a different batch and source of cassava leaves as well as quantifying this using the well-established König's reaction in parallel (Supplementary Information Figs. 8 and 9). This confirmed that the results are reproducible and that in complex media the visual detection limit is reduced to ∼10 μg/mL if 30 μL of sample is added. Moreover, the König's reaction results independently confirmed the cyanide levels in cassava leaves are ∼10 μg/mL from our processing methods. These promising results suggest our ninhydrin paper could be used as a simple in-the-field method to monitor cyanide since solutions of caustic soda can be purchased from shops.

## 3 Conclusion

In summary, we have demonstrated that paper can be impregnated with non-toxic ninhydrin, dried, stored for several weeks and used to semi-quantitatively detect cyanide at concentrations up to ∼5 μg/mL. Through extensive optimisation of the assay, we have shown that the ninhydrin paper preparation is simple (submerge in ninhydrin dissolved in acetone and left to dry for ∼1 h), and that sample processing prior to addition to the paper is minimal (mix with 0.1 M NaOH). A simple pale yellow to red colour change is observed in the presence of cyanide and the colour turns purple after several hours. Most importantly, the assay can be used to monitor cyanide in real samples such as cassava leaves, raising the possibility of the assay being used in-the-field due to the cheap and non-toxic reagents required.

## 4 Experimental

### 4.1 Chemicals and reagents

All chemicals were purchased in high purity (>98 %) from commercial suppliers. The paper used in this work was Whatman paper No.1 (Cytiva, cat. no. 1001-125).

### 4.2 Preparation of ninhydrin paper

Ninhydrin was dissolved in either acetone or aqueous NaOH (0.1 M) at various concentrations (10, 20, 40, 60, 80 and 100 mg/mL; subsequently 100 mg/mL was used for other experiments). Whatman paper No. 1 was then placed into the solution (5 mL) and allowed to absorb the ninhydrin from the solution. For NaOH paper, after ∼5 min, the paper was removed from the solution and allowed to dry at room temperature on the bench overnight. For acetone paper, the solution was allowed to evaporate over ∼1 h. The dried paper was then cut into 1×1 cm pieces and stored in a dark bottle prior to use.

### 4.3 Evaluating cyanide detection using ninhydrin paper

Stock solutions of cyanide (10 mg/mL) were made by dissolving KCN in unbuffered water, NaOH (*aq.*, 0.1 M) or Na_2_CO_3_ (*aq.*, 5% w/v). The stock solutions were serially diluted to the final concentrations (0.1, 1, 5, 10 and 50 μg/mL) using the same solution that was used to make the stock solution. To evaluate cyanide detection, the diluted cyanide solutions (30 μL) were added to the ninhydrin paper. The paper was imaged before any addition (negative control), immediately after addition (0 min) as well as at 5 min, 1 h, 2 h and 24 h (next day) time points. Earlier time points when the paper was still wet (up to 5 min) were recorded using either an iPad Air 2021 or an iPhone 14 Pro, while later time points were scanned using either an EPSON L565 printer (model C463C) or Brother DCP-L3551CDW Laser Printer.

### 4.4 Evaluating specificity of ninhydrin paper

Various different molecules (KCl, KI, NaH_2_PO_4_, KNO_3_, KCN, KCOOH, NaHSO_3_, NaC_4_H_5_O_6_, NaHCO_3_, Na_2_CO_3_, KSCN, **β**-Mercaptoethanol, arginine, cysteine and bovine serum albumin) were dissolved in an aqueous NaOH (0.1 M) solution to give 10 mg/mL stock solutions. In an analogous way to cyanide detection, these stock solutions were diluted to the final target concentration (50 or 500 μg/mL) before addition of the molecule (30 μL) to the ninhydrin paper prepared using acetone. The paper was then imaged immediately (0 min) as well as at 5 min, 1 h, 2 h and 24 h time points. Earlier time points when the paper was still wet (up to 5 min) were recorded using either an iPad Air 2021 or an iPhone 14 Pro, while later time points were scanned using either an EPSON L565 printer (model C463C) or Brother DCP-L3551CDW Laser Printer.

### 4.5 Real sample analysis

Cassava leaves were obtained from cassava farms in either Khon Kaen or Nakhon Si Thammarat, Thailand. Cassava leaves (20 g) were washed twice with water (50 mL ×2) before being crushed using a pestle and mortar for 15 min. The leaf paste was divided into 2 portions (10 g each) for either washing or boiling treatments.

For washing, crushed leaves (10 g) were soaked in water (50 mL) for 15 min before the entire supernatant was removed and filtered. Some of the supernatant was mixed with aqueous 0.1 M NaOH (1 in 10 dilution) and 30 μL of the sample was added to the ninhydrin paper before being imaged at various time points (immediately [0 min], 5 min, 1 h, 2 h and 24 h; “wash 1×”). This process of soaking, removing the supernatant and analysing some of the supernatant was repeated (to give wash 2× , 3× and 4×) until the ninhydrin paper no longer showed a positive signal for cyanide. The paper was then imaged immediately (0 min), after 5 min, 1 h, 2 h and 24 h. Earlier time points when the paper is still wet (up to 5 min) were recorded using either an iPad Air 2021 or an iPhone 14 Pro, while later time points were scanned using either an EPSON L565 printer (model C463C) or Brother DCP-L3551CDW Laser Printer.

For boiling, crushed leaves (10 g) were processed in the same way as for washing but instead of soaking the crushed leaves at room temperature, the crushed leaves and water were boiled at 90 °C using pre-warmed water. The boiled supernatant was allowed to cool, filtered and then diluted with aqueous 0.1 M NaOH (1 in 10 dilution) before being analysed using the ninhydrin paper.

Finally, supernatants were also analysed using the solution-based ninhydrin method or chloramine-T method to cross-validate the results observed on the paper.

### 4.6 Solution-phase determination of cyanide concentration using ninhydrin

A modified version of a previously protocol reported was followed [24,29]. Ninhydrin solutions were degassed before use and stored in a dark bottle. Cyanide solutions (various concentrations in 0.5 mL unbuffered water) were mixed with aqueous ninhydrin (0.08 M, 0.8 mL) and an aqueous solution of Na_2_CO_3_ (0.4 M, 0.8 mL). The mixture was immediately degassed and incubated at room temperature for 10 min. The solution became red if cyanide was present. Next, an aqueous solution of NaOH (2.5 M, 2.9 mL) was added and the reaction was further incubated for 3 min. This caused the solution to turn blue. UV–vis spectra of the solution were then recorded (300–800 nm) and the absorbance at 598 nm (y-axis) was plotted against the final concentration of cyanide in the reaction (x-axis). The data was fitted to the linear equation y=mx+c to create a calibration curve using scipy stats linregress, which was then used to determine the concentration of cyanide from cassava leave washing/boiling solutions.

### 4.7 Solution-phase determination of cyanide concentration using chloramine-T

A modified version of a previously protocol reported was followed [30]. A chloramine-T solution (0.5 w/v) was prepared by dissolving chloramine-T (50 mg) in deionised water (10 mL). A barbituric acid–pyridine mixture was prepared by mixing barbituric acid (0.1948 g), pyridine (1 mL) and water (enough to reach a final total volume of 10 mL). Cyanide solutions (various concentrations in 50 μL phosphate buffered saline, pH 7.4) were mixed with phosphate buffered saline (100 μL, pH 7.4) and water (700 μL). Then the chloramine-T solution (0.5 w/v, 50 μL) was added and immediately after the barbituric acid–pyridine mixture was added (100 μL) and thoroughly mixed. The solution rapidly turned purple. UV–vis spectra of the solution were then recorded (400–700 nm) and the absorbance at 580 nm (y-axis) was plotted against the cyanide concentration in the reaction (x-axis) after subtraction of the zero concentration cyanide blank. The data was fitted to the linear equation y=mx to create a calibration curve, which was then used to determine the concentration of cyanide from cassava leave washing/boiling solutions.

## CRediT authorship contribution statement

**Syafril Hidayat:** Writing – original draft, Methodology, Investigation, Formal analysis, Data curation. **Rachadaporn Benchawattananon:** Supervision, Investigation. **Lapatrada Taemaitree:** Writing – review & editing, Visualization, Supervision, Methodology, Formal analysis, Data curation, Conceptualization.

## Data availability

Data will be made available on request. For requesting data, please write to the corresponding author.

## Declaration of competing interest

The authors declare that they have no known competing financial interests or personal relationships that could have appeared to influence the work reported in this paper.
