# Supplementary material for: Visual detection of cyanide using ninhydrin coated paper
Source: Heliyon. 2025 Jan 24;11(3):e42283. doi: 10.1016/j.heliyon.2025.e42283 (PMC11840534; doi:10.1016/j.heliyon.2025.e42283)
Supplement: Multimedia component 1 [file mmc1.pdf]

Supplementary Information

Visual detection of cyanide using ninhydrin coated paper

Syafril Hidayat[1], Rachadaporn Benchawattananon[1] and Lapatrada Taemaitree[1*]

[1] Department of Integrated Science, Forensic Science Program,
Faculty of Science, Khon Kaen University, Khon Kaen, 40002, Thailand

**Corresponding author. Email: lapata@kku.ac.th*

Table 1. Summary of paper-based detection systems for cyanide including ninhydrin paper method.

	Reagents	Detection	Limitations
**1 Cyantesmo**[1]	• Concentrated sulfuric acid<br>• Paper strip	• Sensitivity (manufacturer): 0.2 µg/mL (10 mL after 15 min; 2 µg)<br>• Sensitivity (found): 0.2 µg/mL (10 mL after 30 min; 2 µg)<br>• Colour: pale blue → dark blue	• Sample must be acidified to release HCN gas (dangerous at higher cyanide concentrations)<br>• Longer times (potentially overnight) for low concentration detection
**2 Quantofix**[1] (based on König's reaction)	• Proprietary phosphate buffer (powder)<br>• Chloramine-T / pyridine solution	• Sensitivity (manufacturer): 1 µg/mL<br>• Sensitivity (found): 4 µg/mL (5 mL after ~1 min; 20 µg)<br>• Colour: white → purple	• Toxic, flammable and irritant reagents required<br>• Multi-step process (sample prep. to below pH 10, add powder then add chloramine-T solution)<br>• SCN^- / Br^- / I^- interfere at concentrations above 1, 5 and 5 µg/mL respectively
**3 Visocolor Eco**[1] (based on König's reaction)	• Chloramine-T solution<br>• Basic isonicotinic acid and 1,3-dimethylbarbituric acid powder<br>• Citrate buffer solution	• Sensitivity (manufacturer): 0.01 µg/mL<br>• Sensitivity (found): 0.05 µg/mL (5 mL after ~15 min; 0.25 µg)<br>• Colour: yellow → blue	• Toxic and irritant reagents required<br>• Multi-step (sample prep., add powder then add chloramine-T solution)<br>• SCN^- / Br^- / I^- interfere at concentrations above 0.1 µg/mL
**4 Picrate paper**[2]	• Plant sample analysis required either enzymes that digest cyanogenic glucoside of interest or 0.1 M phosphoric acid to release gaseous HCN<br>• Picrate embedded paper	• Sensitivity (found): 5 µg/mL (0.5 mL after ~16 h at 25 – 35 °C; 2.5 µg)<br>• Colour: yellow → dark brown	• Acidified paper may release picric acid which is explosive if dry<br>• Reaction times are long<br>• Colour change close on colour wheel<br>• Paper must be suspended in above solution in sealed tube
**5 Origami paper**[3]	• $[Pt(p\text{-}MeC_6H_4)_2(phen)]$ receptor (not commercially available)<br>• Small (~1 µL) volumes of borate buffer and 25% w/v trichloroacetic acid	• Sensitivity (found): 0.02 µg/mL (2 µL after ~1 min; 0.00004 µg)<br>• Colour: white → yellow	• Non-commercial reagents and fabrication methods<br>• Calibration under optimal conditions required
**6 Ninhydrin paper** (current manuscript)	• Ninhydrin coated Whatman paper<br>• 0.1 M NaOH solution	• Sensitivity (found): ~5 µg/mL (30 µL after immediate addition, stable after 24 h; 0.15 µg)<br>• Colour: yellow → red (short times) / purple (long times)	• Uneven colour change on addition of cyanide containing samples<br>• Less sensitive (30 µL of ~10 µg/mL) in more complex media (e.g. crushed cassava leaf extracts)

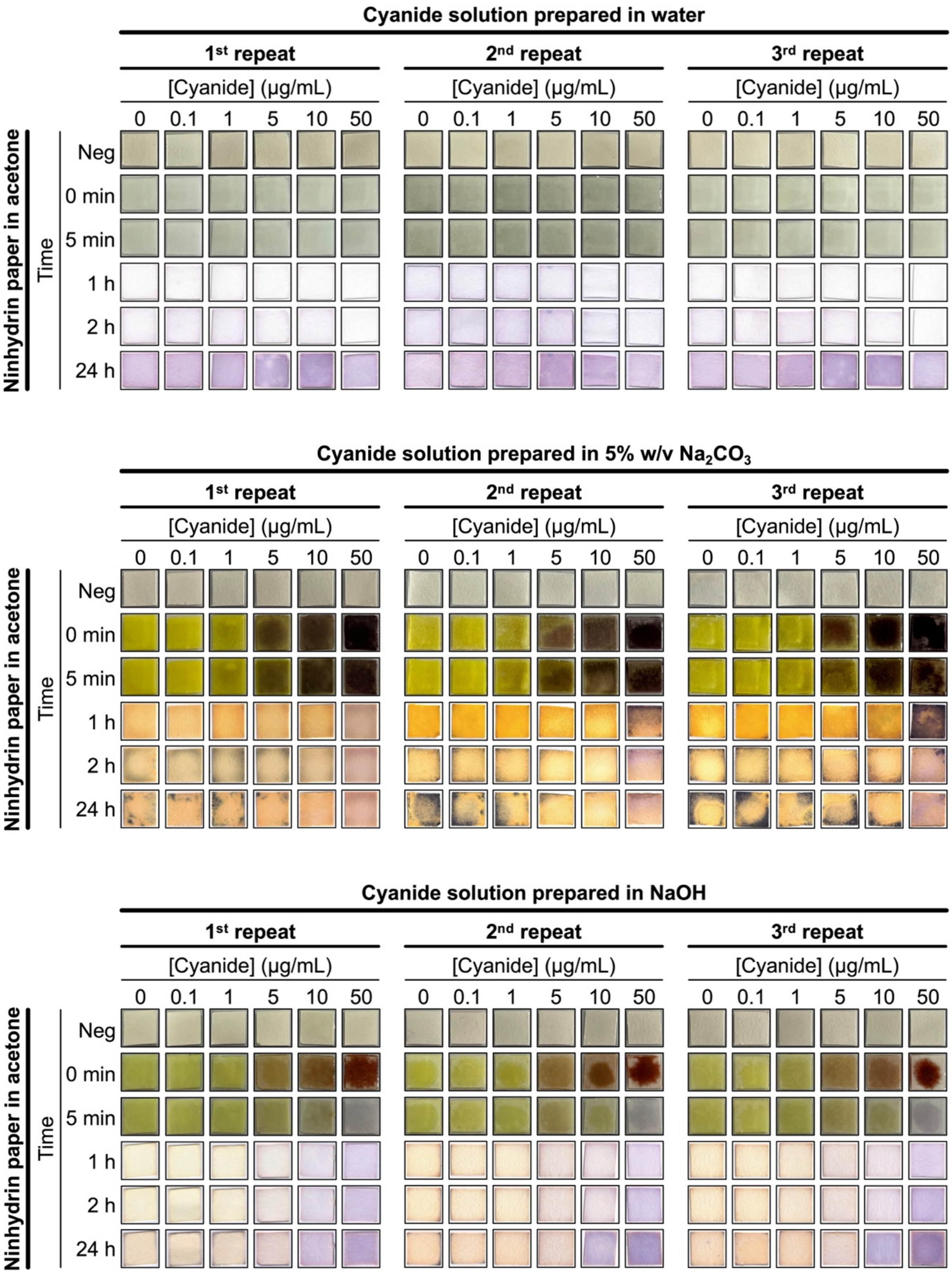
Cyanide solution prepared in water
1st repeat
2nd repeat
3rd repeat
[Cyanide] (μg/mL)
0 0.1 1 5 10 50
Ninhydrin paper in acetone
Time
Neg
0 min
5 min
1 h
2 h
24 h
Cyanide solution prepared in 5% w/v Na2CO3
1st repeat
2nd repeat
3rd repeat
[Cyanide] (μg/mL)
0 0.1 1 5 10 50
Ninhydrin paper in acetone
Time
Neg
0 min
5 min
1 h
2 h
24 h
Cyanide solution prepared in NaOH
1st repeat
2nd repeat
3rd repeat
[Cyanide] (μg/mL)
0 0.1 1 5 10 50
Ninhydrin paper in acetone
Time
Neg
0 min
5 min
1 h
2 h
24 h

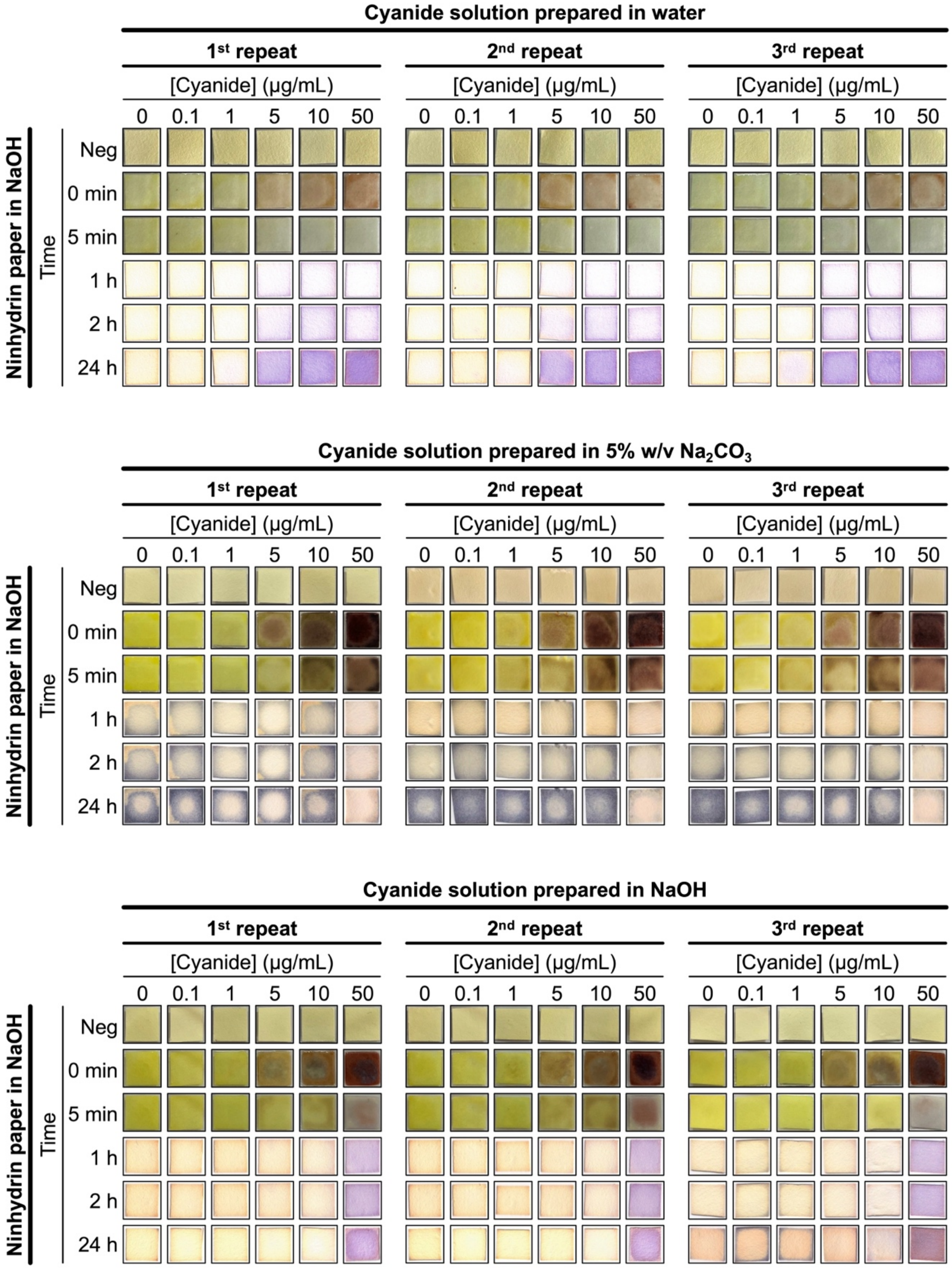

Supplementary Figure 1. Repeat 2 and 3 of data from main Figure 2. Optimisation of the cyanide detection on ninhydrin paper. The Whatman paper was submerged in either an acetone or a 0.1 M NaOH solution of ninhydrin and allowed to dry. Potassium cyanide (0, 0.1, 1, 5, 10 and 50 µg/mL) prepared in either 0.1 M NaOH, 5% w/v Na_2CO_3 or unbuffered water was added to the paper. Colour changes were recorded before (negative control) and after addition of the cyanide solutions to the paper (immediately [0 min], 5 min, 1 h, 2 h and 24 h).

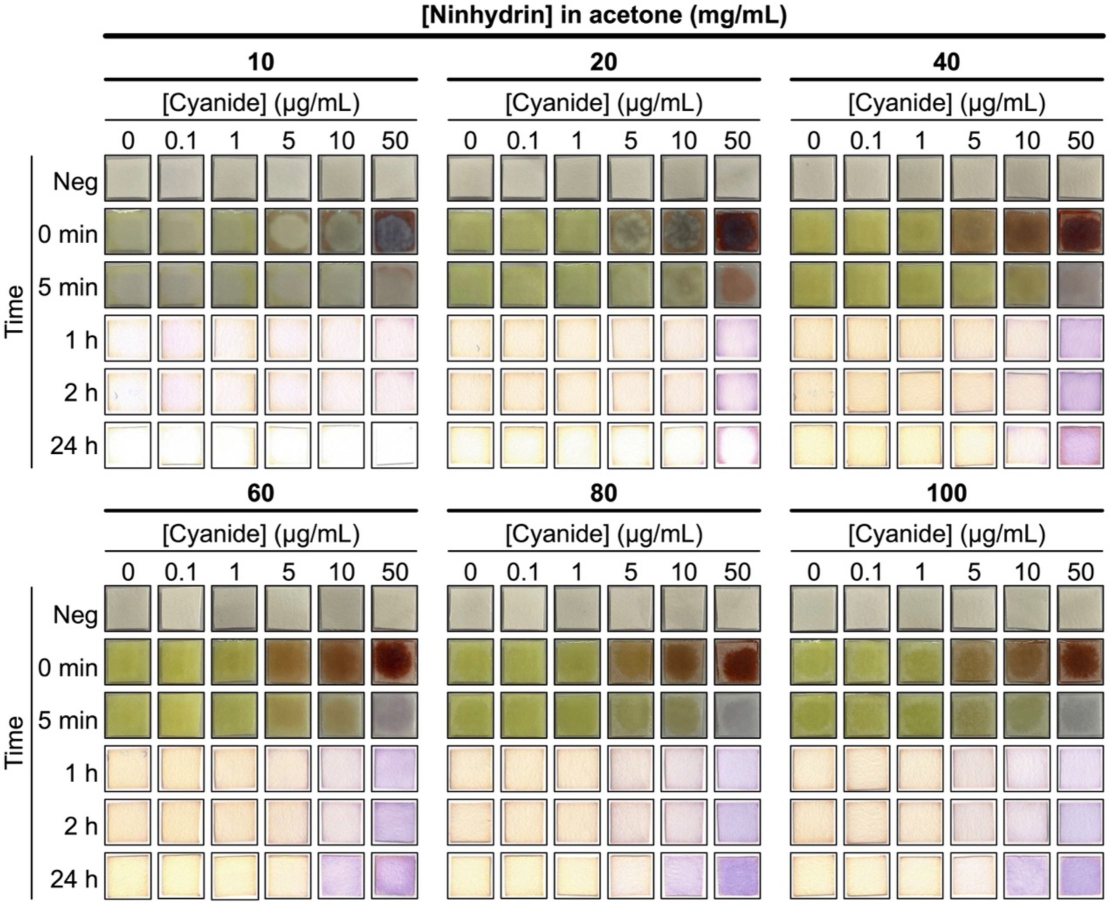

Supplementary Figure 2. The effect of ninhydrin concentration (in acetone) used to prepare the Whatman paper. Concentrations above 40 mg/mL gave consistent results. Potassium cyanide (0, 0.1, 1, 5, 10 and 50 μg/mL) is dissolved in 0.1 M NaOH. Colour changes were recorded before (negative control) and after addition of the cyanide solutions to the paper (immediately [0 min], 5 min, 1 h, 2 h and 24 h).

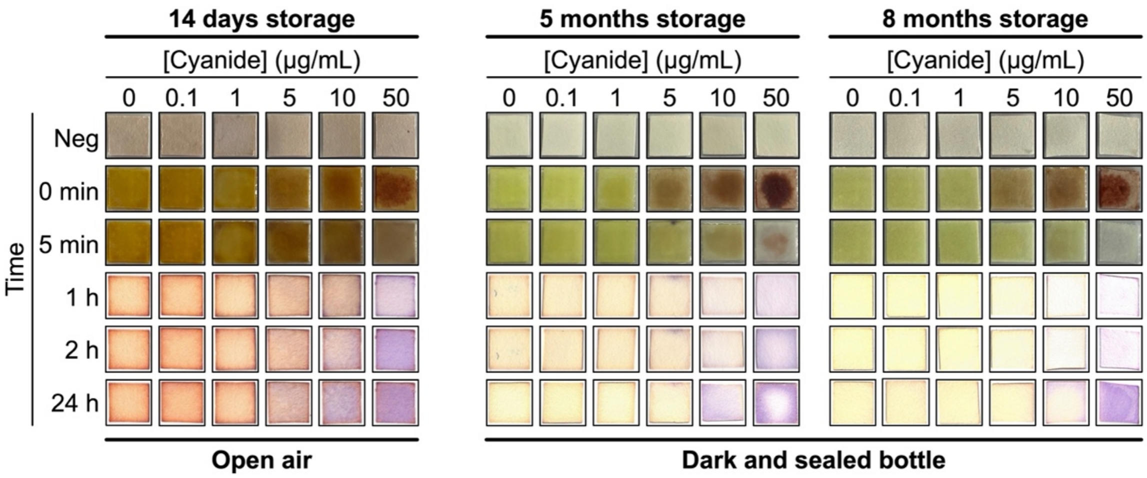

Supplementary Figure 3. Long term storage (14 days, 5 months and 8 months) of the ninhydrin paper (prepared using an acetone solution) in a dark bottle showed minimal differences in detection of cyanide (0, 0.1, 1, 5, 10 and 50 μg/mL) that is dissolved in 0.1 M NaOH compared to freshly prepared paper (see Figure 1 and Supplementary Figure 1). Colour changes were recorded before (negative control) and after addition of the cyanide solutions to the paper (immediately [0 min], 5 min, 1 h, 2 h and 24 h).

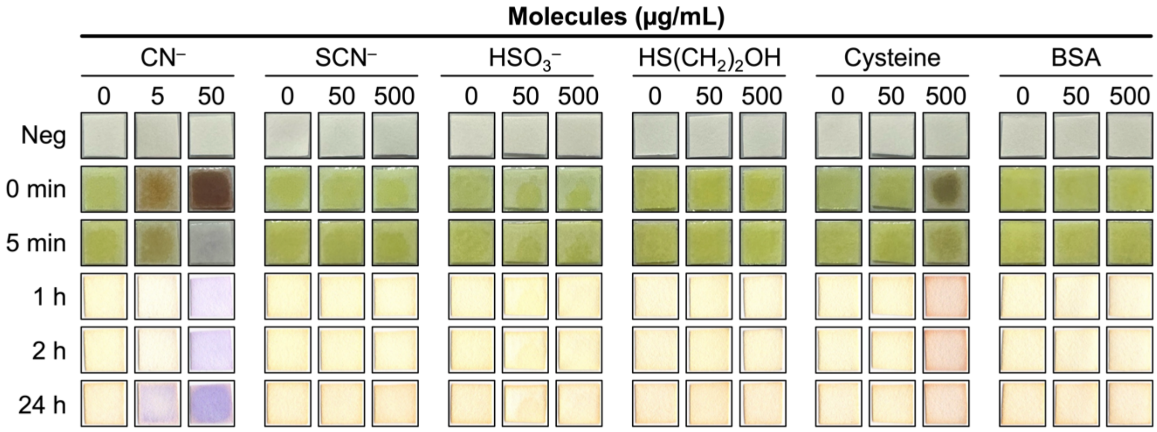

Supplementary Figure 4. Ninhydrin paper is specific for cyanide over sulfer containing molecules (HSO_3^-, SCN^-, $HS(CH_2)_2OH$, cysteine and BSA). Volume added = 30 μL. Colour changes were recorded immediately (0 min), 5 min, 1 h, 2 h and 24 h after addition of each solution onto the paper. Note all molecules are dissolved in 0.1 M NaOH. $HS(CH_2)_2OH$ = **β**-Mercaptoethanol.

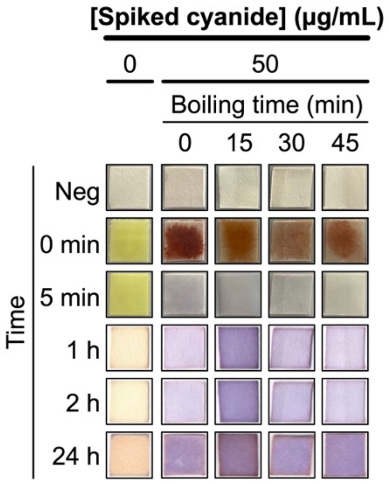

Supplementary Figure 5. Spiking of cassava extracts with 50 µg/mL of potassium cyanide shows the expected colour changes immediately after addition (0 min) as well as 5 min, 1 h, 2 h and 24 after addition. Cassava leaves were boiled for 15 min, 30 min and 45 min. The solution spiked with 50 µg/mL of potassium cyanide and then added to the ninhydrin paper. Colour changes were recorded before (negative control) and after addition of the cyanide solutions to the paper (immediately [0 min], 5 min, 1 h, 2 h and 24 h). Cassava leaves were obtained from Khon Kaen, Thailand.

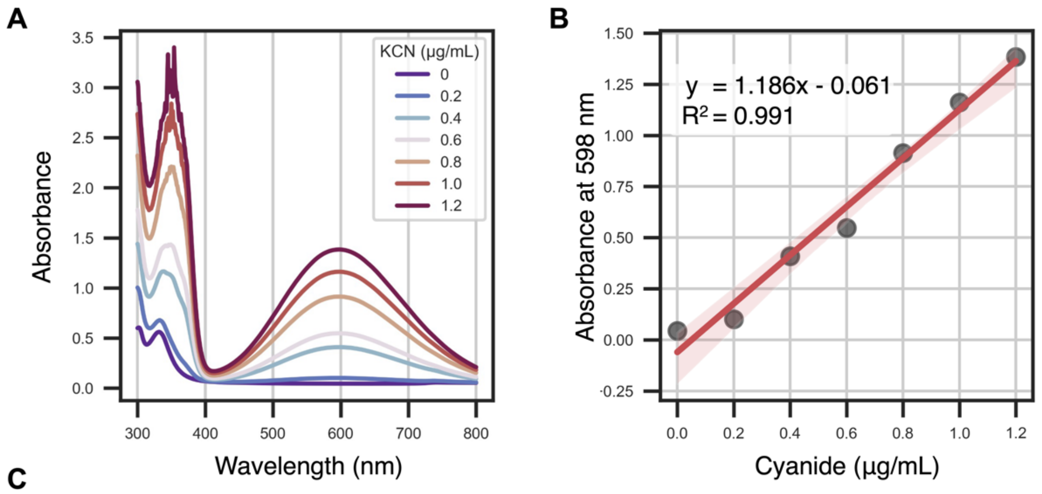

C

	Washing				Boiling		
**Repeat 1**	1×	2×	3×	4×	15 min	30 min	45 min
Absorbance (raw)	0.21	0.28	0.56	0.43	0.22	0.64	0.66
Absorbance (corr.)	21.11	13.86	11.18	2.13	21.51	12.88	1.65
Cyanide (μg/mL)	17.85	11.74	9.48	1.85	18.19	10.91	1.44
**Repeat 2**							
Absorbance (raw)	0.17	0.26	0.45	1.17	0.25	0.54	1.51
Absorbance (corr.)	17.34	12.79	8.92	5.84	25.12	10.74	3.77
Cyanide (μg/mL)	14.67	10.84	7.57	4.98	21.23	9.11	3.23

Supplementary Figure 6. Solution phase ninhydrin analysis of cyanide in cassava leaves for Figure 4 and Supplementary Figure 6. (A) shows the standard curve absorbance spectra of known cyanide concentrations in a ninhydrin solution. (B) shows the standard curve from plotting the absorbance at a wavelength of 598 nm (y-axis) against standard cyanide concentrations (x-axis). (C) shows tabulated raw absorbances, the values once corrected for dilution (to ensure they are in the linear range of the machine's optics, corr.) and the final calculated concentrations based on the standard curve linear fit. Cassava leaves were obtained from Khon Kaen, Thailand.

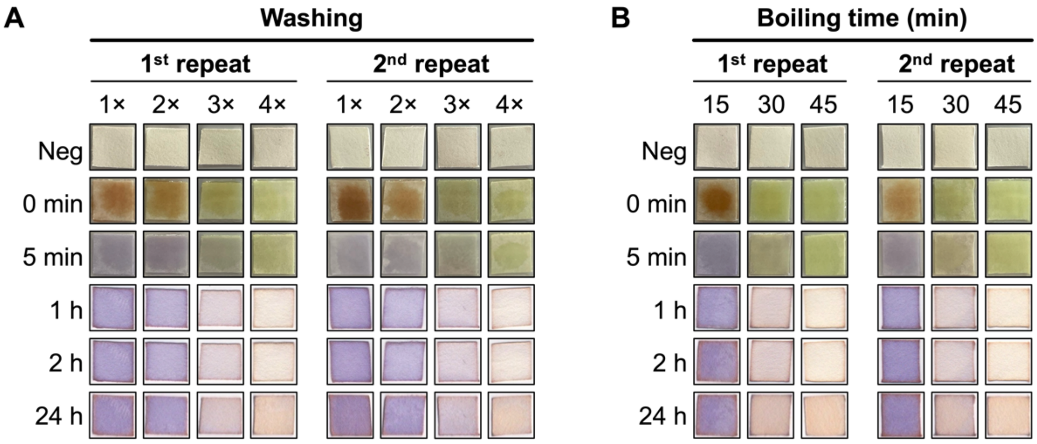

Supplementary Figure 7. An additional repeat for main Figure 4. Detecting cyanide in processed cassava leaves using ninhydrin paper. (A) Cassava leaves were washed first time (1×), second time (2×), third time (3×) and forth time (4×). As expected, more washes with water results in lower cyanide concentrations. (B) Cassava leaves were boiled for 15 min, 30 min and 45 min. The solution was added to the ninhydrin paper at each time point. The longer the boiling, the lower the concentration of cyanide remained. Cassava leaves were obtained from Khon Kaen, Thailand.

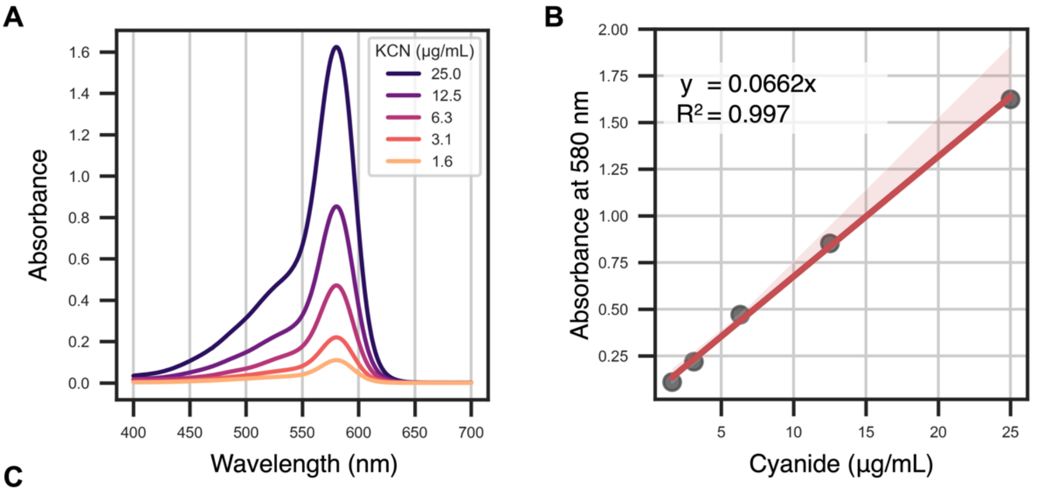

C

	Washing				Boiling			
**Repeat 1**	1×	2×	3×	4×	15 min	30 min	45 min	60 min
Absorbance	0.746	0.463	0.231	0.152	0.680	0.161	0.057	0.021
Cyanide (μg/mL)	11.27	6.99	3.48	2.30	10.27	2.44	0.86	0.31
**Repeat 2**								
Absorbance	0.878	0.442	0.247	0.141	0.732	0.202	0.062	0.025
Cyanide (μg/mL)	13.26	6.68	3.72	2.13	11.05	3.05	0.94	0.38
**Repeat 3**								
Absorbance	0.788	0.363	0.177	0.128	0.514	0.183	0.063	0.028
Cyanide (μg/mL)	11.90	5.48	2.67	1.94	7.77	2.77	0.95	0.43

Supplementary Figure 8. Solution phase chloramine-T analysis of cyanide in cassava leaves for Supplementary Figure 9. (A) shows representative standard curve absorbance spectra of known cyanide concentrations in a chloramine-T solution. (B) shows a representative standard curve from plotting the absorbance at a wavelength of 580 nm (y-axis) against standard cyanide concentrations (x-axis). (C) shows tabulated absorbances and the final calculated concentrations based on the standard curve linear fit. Cassava leaves were obtained from Nakhon Si Thammarat, Thailand.

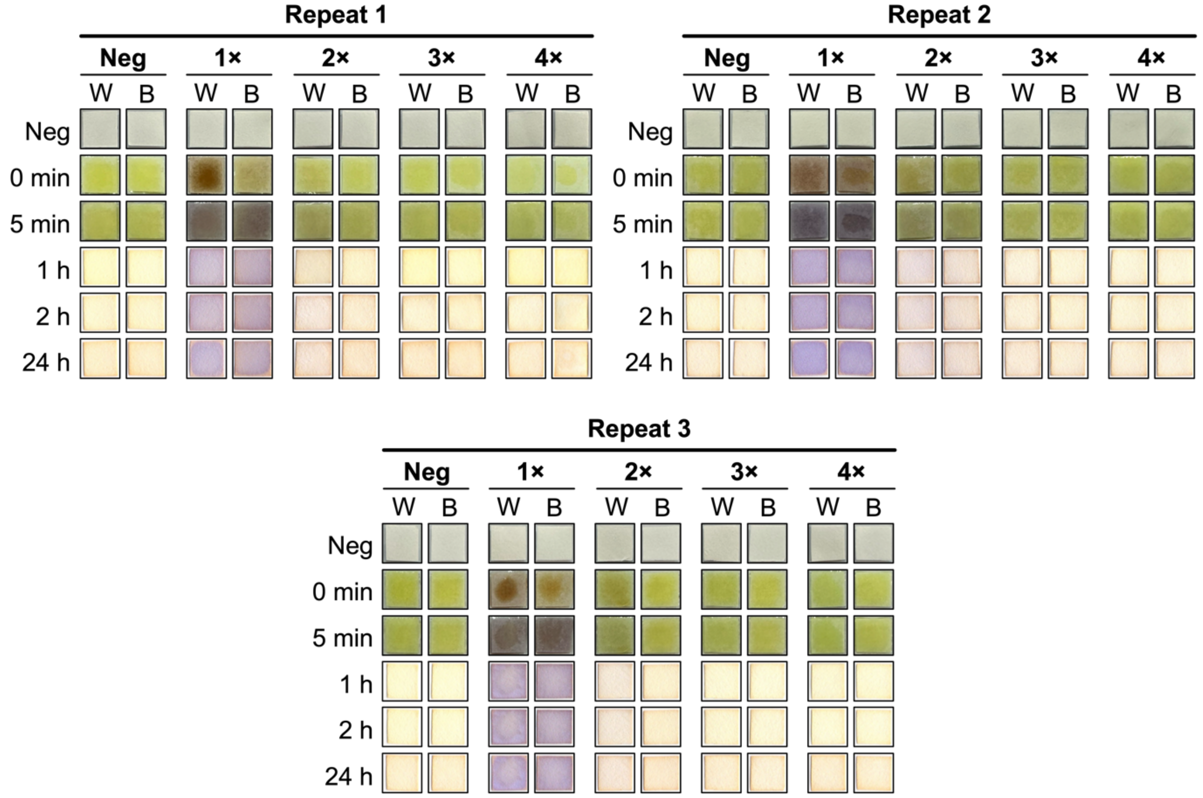

Supplementary Figure 9. Detecting cyanide in processed cassava leaves using ninhydrin paper. These samples were also used in chloramine-T measurements in Supplementary Figure 8. W = wash. B = boil. Neg. (column) = 0.1 M NaOH only. Neg. (row) = before sample addition. 1× = after first 15 min of the specified treatment (either wash or boil). 2× = after second 15 min of the specified treatment. 3× = after third 15 min of the specified treatment. 4× = after fourth 15 min of the specified treatment. The longer the boiling or washing times, the lower the concentration of cyanide remained. Cassava leaves were obtained from Nakhon Si Thammarat, Thailand.

References

(1) Hicklin, D. W.; Willingham, W. A.; States, A. E. C. B. C. A. P. G. U. *Evaluation of Commercially Available Cyanide Test Kits against Various Matrices*; Tech. rep. Army Edgewood Chemical Biological Center Aberdeen Proving Ground ..., 2016. https://apps.dtic.mil/sti/citations/AD1014443 (accessed 2024-08-05).

(2) Rezaul Haque, M.; Howard Bradbury, J. Total Cyanide Determination of Plants and Foods Using the Picrate and Acid Hydrolysis Methods. *Food Chem* **2002**, *77* (1), 107–114. https://doi.org/https://doi.org/10.1016/S0308-8146(01)00313-2.

(3) Sheini, A.; Aseman, M. D.; Bordbar, M. M. Origami Paper Analytical Assay Based on Metal Complex Sensor for Rapid Determination of Blood Cyanide Concentration in Fire Survivors. *Sci Rep* **2021**, *11* (1), 3521. https://doi.org/10.1038/s41598-021-83186-0.
